# Identification of potential extracellular signal-regulated protein kinase 2 inhibitors based on multiple virtual screening strategies

**DOI:** 10.3389/fphar.2022.1077550

**Published:** 2022-11-18

**Authors:** Ruoqi Yang, Guiping Zhao, Lili Zhang, Yu Xia, Huijuan Yu, Bin Yan, Bin Cheng

**Affiliations:** ^1^ Department of Acupuncture, Affiliated Hospital of Shandong University of Traditional Chinese Medicine, Jinan, China; ^2^ School of Pharmacy, Shandong University of Traditional Chinese Medicine, Jinan, China; ^3^ Institute of Chinese Materia Medica, China Academy of Chinese Medical Sciences, Beijing, China; ^4^ Department of Ultrasound, Jinan Central Hospital Affiliated to Shandong First Medical University, Jinan, China

**Keywords:** extracellular signal-regulated protein kinase 2, virtual screening, inhibitors, machine learning, natural products

## Abstract

The integration of multiple virtual screening strategies facilitates the balance of computational efficiency and prediction accuracy. In this study, we constructed an efficient and reliable “multi-stage virtual screening-in vitro biological validation” system to identify potential inhibitors targeting extracellular signal-regulated protein kinase 2 (ERK2). Firstly, we rapidly obtained 10 candidate ERK2 inhibitors with desirable pharmacokinetic characteristics from thousands of named natural products in ZINC database based on machine learning classification models and ADME/T prediction. The structure-based molecular docking approach was then used to obtain four further hits with lower binding free energy compared to the positive control molecule Magnolipin. Subsequently, the two compounds were purchased for *in vitro* biological validation considering commercial availability and economic cost, and the results showed that Dodoviscin A exhibited acceptable inhibitory activity on ERK2 (IC_50_ = 10.79 μm). Finally, the mechanism of action and binding stability of this natural product inhibitor were investigated by binding mode analysis and molecular dynamics simulation.

## 1 Introduction

Overexpression of kinases is a major contributor to the pathogenesis of many diseases, such as cancer, inflammation and neurodegenerative diseases. The search for inhibitors that target kinases has therefore become one of the hot topics in drug discovery (Gharwan and Groninger, 2016). The mitogen-activated protein kinase (MAPK) family consists of serine/threonine kinases that are widely expressed in many cellular tissues and are implicated in a variety of cellular processes including cell growth, differentiation and apoptosis (Dhillon et al., 2007). Four parallel MAPK signaling pathways have been identified, of which the extracellular signal-regulated protein kinase 1/2 (ERK1/2) pathway has been the most extensively studied. In this pathway, extracellular signals (e.g., epidermal growth factor) first bind to the receptor tyrosine kinase (RTK), which subsequently stimulates the conversion of Ras proteins from GDP-binding to GTP-binding, thereby activating Raf proteins; and then the activated Raf proteins are involved in catalyzing the phosphorylation of MEK1/2, which further catalyzes the phosphorylation of ERK1/2 (Chang and Karin, 2001).

Many inhibitors against upstream targets achieved good clinical efficacy initially, but they all developed resistance after a few months, the main reason for this being the reactivation of the downstream target ERK (Pratilas and Solit, 2010). Many studies have shown that inhibitors against ERK2 are more specific and have a relatively low probability of acquiring resistance, making ERK2 an ideal therapeutic target (Carlino et al., 2014). Like other protein kinases, the primary structure of ERK2 consists of an N-terminal and a C-terminal structural domain, the former containing five antiparallel β-sheet structures (β1 ∼ β5), an αC-helix structure and a glycine-rich loop, while the latter containing six conserved α-helix structures and four shorter β-sheet structures (β6 ∼ β9). Besides, there is an ATP-binding pocket in the hinge region connecting the two, which is the binding site for most current kinase inhibitors (Lefloch et al., 2008).

The high diversity of chemical structures and physicochemical properties of natural products makes them a valuable source for the discovery of novel active compounds with representative success stories including pilocarpine, morphine, and artemisinin (Zhang et al., 2020). Compared to synthetic compounds, natural products are widely available, inexpensive and have lower toxic effects. Unfortunately, there are still relatively few reports of natural products targeting kinases, which means that the search for natural products with ERK2 inhibitory activity is a valuable direction to explore. In the pharmaceutical industry, the discovery and experimental validation of active compounds is a time-consuming and laborious process. Traditional high-throughput screening often requires biochemical testing of over a million compounds individually to identify the active ingredients, which is costly in terms of time and money. In contrast, virtual screening techniques can significantly reduce the number of compounds used for pharmacological activity testing, resulting in significant cost savings (Gupta et al., 2021).

**FIGURE 1:**
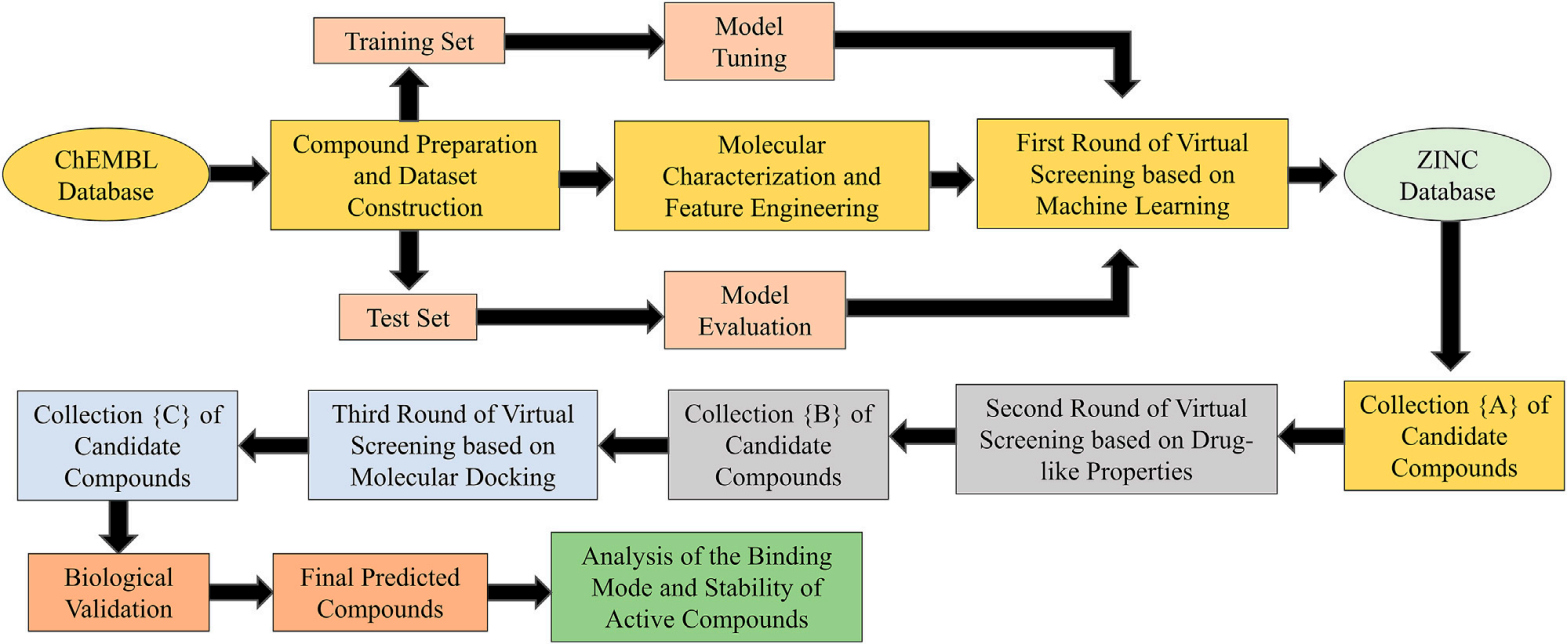
Architecture of the sequential virtual screening process.

With the rapid development of medical research, increasing experimental data on drugs is available to researchers. In this context, using the wealth of results from the pharmaceutical field to find target compounds is a more accurate and cost-effective way of drug discovery (Paul et al., 2021). With the booming era of big data, artificial intelligence technologies, represented by machine learning, have made significant progress in shortening the drug development cycle, which provides strong support for the virtual screening of lead compounds. In many cases, machine learning exhibits superior performance compared to traditional computational methods and has unique advantages in the screening of compounds (Tsou et al., 2020). Based on the research background, we have designed a “sequential” virtual screening process that combines machine learning with multiple cheminformatics tools to identify natural product inhibitors targeting ERK2, and we have also performed *in vitro* biological evaluation of the screened compounds to validate the reliability of the process. The technical route involved in this study was shown in Figure 1.

## 2 Materials and methods

### 2.1 Data pre-processing, molecular characterization, and feature engineering

In the present study, experimental IC_50_ values for ERK2 inhibitors were collected from the ChEMBL database. Due to some variation between data from different sources, we handled duplicate compounds according to the following criteria: 1) If a compound had multiple identical IC_50_ values, only one of them was retained. 2) If a compound had multiple different IC_50_ values, the average of them was used as the final IC_50_ value. In addition, if a compound had no clear IC_50_ value, it was excluded. Next, 422 compounds were labelled as “active” (indicated by number 0) and 442 compounds as “inactive” (indicated by number 1) based on an activity cut-off value of 1 μm. Finally, the entire data set was randomly divided into a training set and a test set in the ratio of 8:2. The above process was completed by writing code in Python 3.7.

The input characterization of compounds in the dataset takes the form of molecular descriptors and molecular fingerprints. Molecular descriptors are numerical representations of chemical and biological properties and are classified into different types such as topological, compositional and geometric descriptors (Wei et al., 2021). Molecular fingerprints encode structural information in the form of a sequence of binary bits, with the corresponding bit being 1 if a predetermined bond or functional group is present in the molecule and 0 otherwise (Racz et al., 2019). Molecular descriptors were calculated using the cheminformatics toolkit RDKit, containing a total of 208 numerical indicators representing the physicochemical and structural characteristics of the molecule. MACCS molecular fingerprints were calculated using the open source software PaDEL-Descriptor, containing a total of 166 binary numbers representing the presence or absence of specific structural fragments in the molecule.

In general, not all features facilitate the construction of machine learning models. Irrelevant and unnecessary features may reduce the generalization ability of the model and lead to overfitting. Therefore, feature engineering is an integral step. Considering the wide range of values for different molecular representations, the corresponding strategies were adopted: for molecular descriptors, all descriptors with a variance of 0 and those not related to the target value (i.e., whether the compound is active or not) were eliminated by variance filtering and mutual information methods. The final 132 molecular descriptors were retained for the construction of machine learning models. For molecular fingerprints, recursive feature elimination combined with learning curves was used to find the optimal number of features, and 80 features with high relevance to the target value were retained.

### 2.2 Generation and evaluation of machine learning models

Machine learning is an important branch of artificial intelligence and has been widely used in recent years for the discovery of lead compounds and the prediction of physicochemical and biological properties of molecules (Serafim et al., 2020). The decision tree algorithm presents the model building process through a tree-like structure, with the intermediate nodes representing the selected features and the leaf nodes representing the decision results. Random Forest (RF) is an ensemble learning algorithm based on decision trees, which overcomes the shortcomings of single decision trees that are prone to overfitting (Svetnik et al., 2003). The RF algorithm obtains some features randomly from the training set and constructs multiple decision tree models using random sampling to make predictions together. Support Vector Machine (SVM) is a machine learning algorithm based on statistical theory, the core idea of which is to find a hyperplane with a maximum bound to classify the training samples. The kernel function is a unique trick of the SVM algorithm, which solves the problem of indistinguishability by mapping the training samples into a high-dimensional space (Mavroforakis and Theodoridis, 2006). Just as the brain usually obtains information from experience to solve problems, the architecture of an Artificial Neural Network (ANN) consists of multiple interconnected neurons distributed in different layers. ANN first calculates the loss by forward propagation and then updates the weights by backward propagation. This process is iterated until the best weights that minimize the loss of the model are found (Jing et al., 2018). In addition, model fusion can also effectively reduce the prediction error of virtual screening. Voting is a voting-based model fusion strategy, while Stacking is a learning-based model fusion strategy. The above machine learning models were generated with the help of the open source Python toolkit Scikit-learn (Abraham et al., 2014).

We used a 10-fold cross-validation of the training set with the test set to evaluate the prediction performance and generalization ability of the model. Accuracy represents the proportion of samples with correct predictions to the total number of samples, and an excellent machine learning classifier should have an accuracy score close to 1. Precision represents the proportion of samples that are truly positive out of all samples that are predicted to be positive, while recall represents the proportion of samples that are correctly predicted out of all samples that are truly positive. F1-score is the harmonic average of precision and recall, which allows an objective evaluation of the predictive performance in the case of an unbalanced data set. The Area Under Curve (AUC) is one of the most important metrics in the evaluation of machine learning models, with an AUC value closer to 1 meaning that the model is more capable of classification.

### 2.3 Visualization of the chemical spatial distribution

Principal component analysis (PCA) is a common linear dimensionality reduction method whose main purpose is to compress data and remove redundant noise while minimizing the loss of original information. PCA is able to derive a few principal components from the original variables so that they retain as much information as possible and are uncorrelated with each other (Cortés et al., 2017). In this study, we visualized the chemical distribution of compounds in the three-dimensional space by using the decomposition. PCA module of Scikit-learn.

### 2.4 Pharmacokinetic analysis and toxicity assessment

Early prediction of ADME/T (absorption, distribution, metabolism, excretion and toxicity) of lead compounds can be effective in avoiding adverse drug reactions in clinical practice (Lee and Chen, 2019). Many filtering methods based on specific drug-likeness are often used as reference standards for screening desirable compounds and thus guide the decision-making process in drug development. We used the server ADMETlab 2.0 to make fast and accurate online predictions of ADME/T for the compounds obtained from the first round of screening, while filtering out those molecules that did not meet the requirements according to a range of parameters (Xiong et al., 2021).

### 2.5 Molecular docking calculations

Molecular Docking predicts the interaction pattern between a target protein and a candidate compound and calculates the corresponding binding free energy. The theory is based on the fact that the binding process of ligands and receptors depends on the matching of spatial shapes (Ferreira et al., 2015). The crystal structure of the receptor protein used in this study was downloaded from the PDB database (PDB ID: 1TVO) and then preprocessed by AutoDock Tools software as follows: water molecules were removed; hydrogen atoms were added and charges were recalculated (Ohori et al., 2005). In addition, the structure optimization of the target protein was performed with the help of the web server FoldX (Schymkowitz et al., 2005). The structures of the ligand compounds were downloaded from the PubChem database and then energy minimization was performed by Chem3D software. Finally, the structures of both receptor and ligands need to be converted to pdbqt format.

We performed a molecular docking study of the compounds obtained from the second round of screening and the target protein using Autodock Vina software (Trott and Olson, 2010). The parameters of the docking box were set to center_x = 6.429, center_y = -4.372, center_z = 16.444, size_x = size_y = size_z = 10.598, and the number of exhaustiveness was set to 30 to ensure the accuracy of the prediction results. After docking was completed, we visualized the docking conformation of the ligand at the active site of the target protein with the help of LigPlot+ and PyMOL software (Laskowski and Swindells, 2011).

### 2.6 Kinase activity assay

The inhibitory activity of the candidate compounds against ERK2 kinase was assayed by the Lance^®^ Ultra kinase assay. The principle of this method is that when a ULightTM-labeled peptide substrate is phosphorylated by the kinase, its phosphorylation site is recognized by the fluorescent europium (Eu)-labeled monoclonal antibody and the energy transfer that occurs during the process is captured by the instrument (Townsend et al., 2012).

In this study, the initial concentration of the candidate compounds purchased from Macklin (Shanghai, China) was set at 100 µm and the gradient dilution in the 384-well plate was set to 2-fold. Staurosporine purchased from MedChemExpress (New Jersey, United States) was used as a positive control (Meggio et al., 1995). Firstly, 40 µl of the test compound, 10 µl of the kinase solution and 10 µl of the substrate solution containing ATP were incubated at room temperature for 60 min (reaction step). At the end of the reaction, EDTA was added to stop the reaction. Then 20 µl of the detection solution containing the antibody dilution was added to the well plate and incubated at room temperature for 60 min (detection step). Finally, the IC_50_ values of the candidate compounds were calculated using a dose-effect curve. Each assay was repeated in triplicates and the results were shown as mean ± standard deviation.

### 2.7 Molecular dynamics simulation

Molecular dynamics simulations based on Newtonian mechanics were able to assess the binding stability of candidate compound Dodoviscin A to target protein ERK2 on both temporal and spatial scales, and the known natural product inhibitor Magnolin was used as a control (Wang et al., 2018). In this study, we performed molecular dynamics simulations using Gromacs software (Pronk et al., 2013). The topology file of ligand compound was generated by the online web tool Swiss Param (https://www.swissparam.ch/), and the topology file of target protein was obtained from the pdb2gmx module (the force field was specified as CHARMM 36). The complex was then confined in a water-filled cubic box (distance between the complex and the edge of the box was at least 10 Å) and the charge of the whole system was neutralized by the addition of gegenions (101 Na+ and 97 Cl-). Next, the system was energy minimized at 10 kJ/mol using the steepest descent method of 50,000 steps (treatment of long-range electrostatic interactions was chosen as PME). Before the final simulation, the system was pre-equilibrated to stabilize at a suitable temperature and pressure, where the temperature for NVT equilibration was maintained at 300 K (Velocity-Rescale was set as the temperature control method) and the pressure for NPT equilibration was maintained at 1 bar (Parrinello-Rahman was set as the pressure control method), and the time duration was both set to 5,000 ps? After the above process, a molecular dynamics simulation was performed for 100 ns and repeated three times, with the trajectory recorded in atomic coordinates at an interval of 2 fs?

## 3 Results

### 3.1 Generation and performance evaluation of machine learning models

**FIGURE 2:**
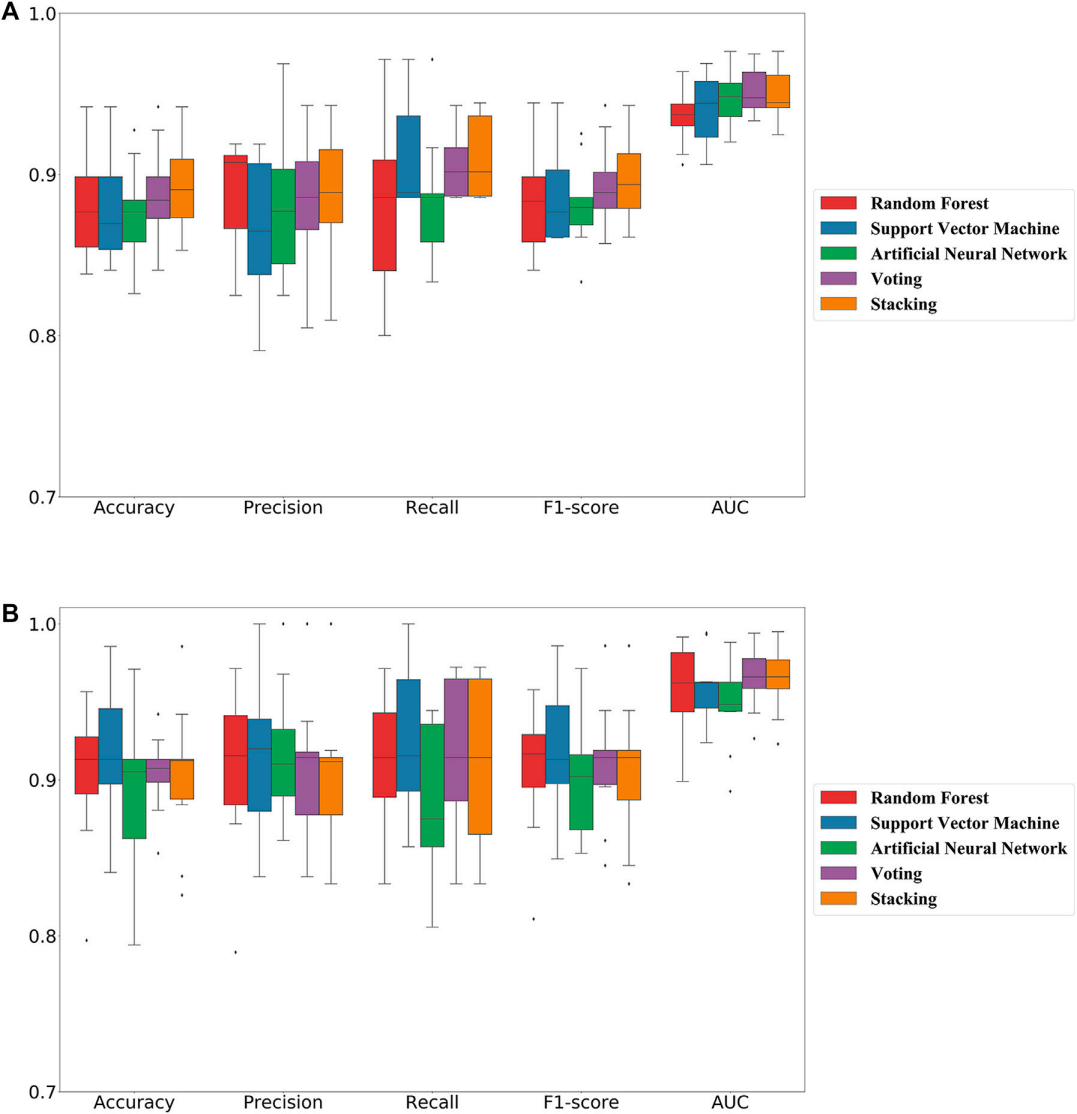
10-fold cross-validation results of five machine learning classification models on the training set. **(A)** Models constructed based on molecular fingerprint; **(B)** Models constructed based on molecular descriptor.

In this study, we adopted popular machine learning techniques for the activity prediction of compounds. The hyperparameters of the model were tuned by the Bayesian Optimization algorithm and a range of metrics of the tuned model were compared on the training and test sets. Cross-validation is one of the most reliable methods for assessing model performance (Fourches et al., 2015). In K-fold cross-validation, the dataset was divided into K subsets, one of which was selected as the validation set for each training epoch, and the remaining (K-1) subsets were used as the training set. This process was repeated K times, so that each subset was used exactly once. Finally, the average of the K training results was used as the final result. The results of the 10-fold cross-validation of the machine learning models generated using the best combination of hyperparameters on the training set were shown in Figure 2. The overall prediction accuracy of the three single models constructed based on MACCS fingerprints ranged from 87.6% to 88.1%, with F1-score values between 0.881–0.887 and AUC values between 0.941–0.949; while the overall prediction accuracy of the three single models constructed based on RDKit descriptors ranged from 89.2% to 91.4%, with F1-score values between 0.895–0.917 and AUC values between 0.948–0.960. The prediction performance of each model on the test set was shown in Table 1, where we found that almost all of these single models outperformed the training set on the test set, while employing RDKit descriptors as a form of molecular representation was a better choice for the ERK2 dataset. It should be noted that according to the spatial distribution of the training set/test set (Supplementary Figure S1), we could observe that the training set almost covered the test set (36.07%, 26.26%, and 13.95% explained variance contribution rate in the three dimensions, respectively). This is also the reason why these models had better performance on the test set.

**TABLE 1:** Comparison of the performance of machine learning classification models based on two forms of molecular representation on the test set.

	Models	Accuracy	Precision	Recall	F1-score	AUC
MACCS Fingerprint	RF	0.913	0.930	0.898	0.913	0.975
	SVM	0.908	0.929	0.887	0.907	0.973
	ANN	0.844	0.886	0.796	0.838	0.930
	Voting	0.873	0.923	0.818	0.868	0.968
	Stacking	0.884	0.925	0.841	0.881	0.970
RDKit Descriptor	RF	0.942	0.954	0.932	0.943	0.979
	SVM	0.936	0.953	0.921	0.936	0.977
	ANN	0.908	0.939	0.875	0.906	0.971
	Voting	0.930	0.952	0.909	0.930	0.980
	Stacking	0.931	0.942	0.921	0.931	0.982

Considering the excellent performance of the above three algorithms on the training and test sets, the model fusion of them is beneficial to integrate the applicability of different algorithms and avoid the local bias of a single model affecting the generalization performance of the prediction. When evaluating binary classification models, AUC is relatively more informative as the most important parameter for measuring model performance. As can be seen in Figure 2; Table 1, the two integrated models (Voting and Stacking) constructed based on RDKit molecular descriptors exhibited AUC values of 0.966 and 0.965 on the training set and 0.980 and 0.982 on the test set, respectively, indicating that performing model fusion produced more desirable classification results and generalization ability. It is worth noting that although a single model could sometimes show better performance in terms of accuracy as well as F1-score, as reported by Gramatica et al., any algorithm has its own priorities and a single model may easily fall into local minima, leading to its poor generalization ability (Gramatica et al., 2007). Therefore, we consider that the two integrated models with the highest AUC values have better predictive reliability.

### 3.2 Virtual screening based on machine learning

**FIGURE 3:**
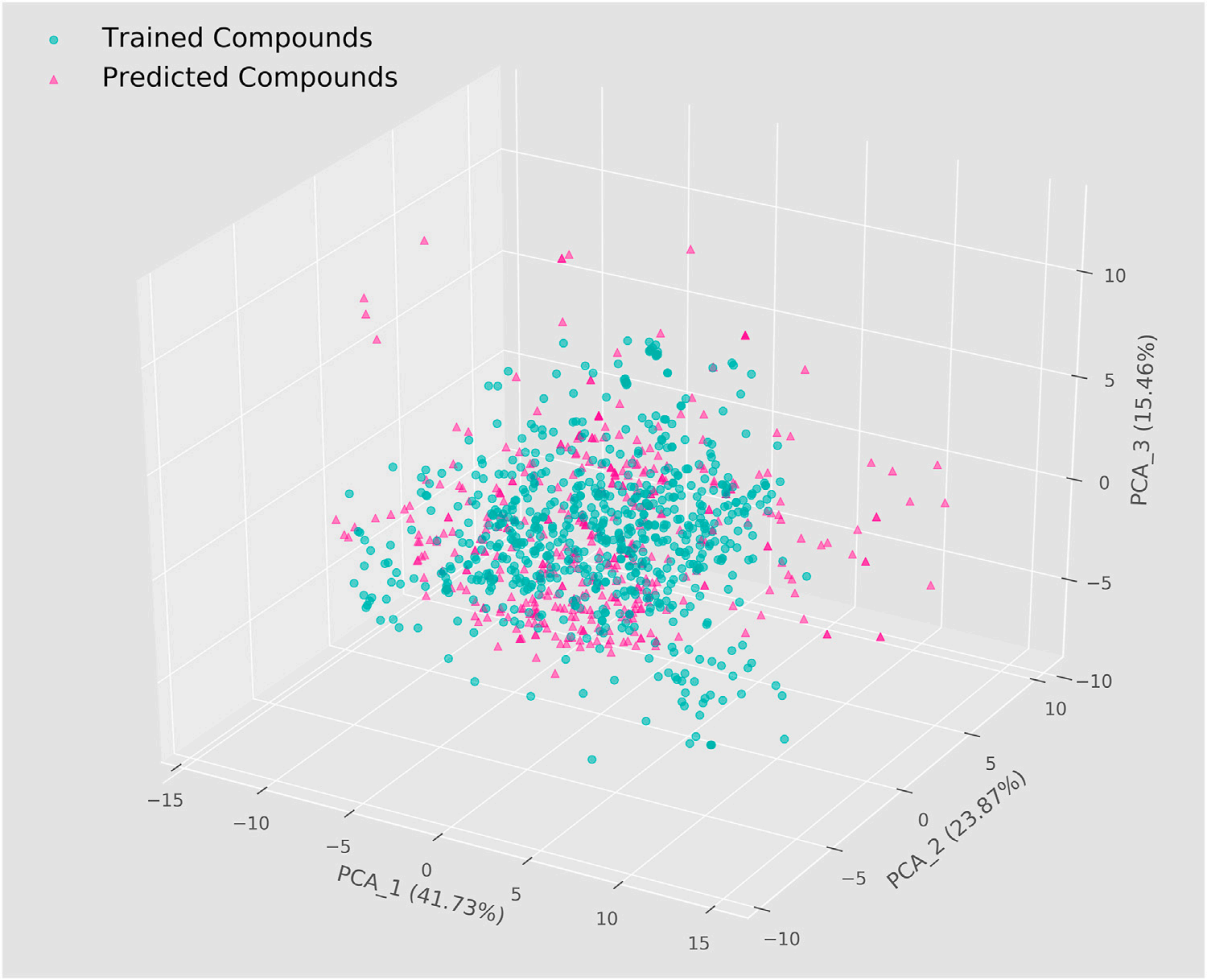
Chemical spatial distribution of trained compounds and predicted compounds.

After generating machine learning models and evaluating their predictive performance, we performed a virtual screening of 4,112 named natural products in the ZINC database using two integrated models based on molecular descriptors. The 208 RDKit molecular descriptors for these compounds were first calculated and then the same data pre-processing and feature selection steps were applied. To improve the reliability of the prediction results and further narrow the scope of the search, we set the threshold for both integrated models to 0.75, which ultimately showed that a total of 427 compounds were predicted as potential ERK2 inhibitors by both models. In general, predictions are reliable when the chemical space of the screened molecules lies in the application domain of the model (Yang et al., 2022). We therefore visualized the training set used for modeling and the corresponding compounds in the ZINC database by means of the PCA algorithm (Figure 3). The results showed that the chemical space distribution of most of the predicted compounds overlapped with that of the compounds in the training set, implying that the virtual screening results based on machine learning were dependable. In addition, the three dimensions could represent 41.73%, 23.87%, and 15.46% of the original data information, respectively, which means that the spatial distribution was of reference value. 403 candidate compounds were selected for subsequent studies following the elimination of certain compounds that were far from the application domain of the model.

### 3.3 Drug-like filtering of potential inhibitors of ERK2 and virtual screening based on molecular docking

Excellent pharmacokinetic properties contribute to reducing the risk of failure of drug candidates in the clinical trial stage. The ADME/T of 403 compounds obtained from the first round of screening was predicted and further filtered according to the set criteria. According to Lipinski’s rule, candidate compounds should satisfy at least three of the five items: Octanol-Water Partition Coefficient (LogP)≤5, Molecular Weight≤500, Number of Hydrogen Bond Acceptors≤10, Number of Hydrogen Bond Donors≤5 and Number of Rotatable Bonds≤10. Drug absorption in the gastrointestinal tract is a key factor in oral drug delivery, and the Human Intestinal Absorption (HIA) rate of a candidate compound should be greater than 30%. Blood-brain barrier (BBB) permeability indicates the effect of the drug on the central nervous system, and an ideal ERK2 inhibitor should have the ability to penetrate the BBB. Water Solubility (LogS) is one of the criteria for molecular absorption, with a reasonable range between −4 and 0.5. In addition, the assessment of the toxicity risk of candidate compounds is more essential in the early stages of drug discovery. All compounds should not show risks of Hepatotoxicity, Acute Oral Toxicity, Carcinogenicity and AMES Mutagenicity. The above criteria resulted in 10 drug-like compounds with desirable ADME/T properties, which would be used in molecular docking studies to compare the binding affinity to ERK2. Partial physicochemical properties of these compounds were listed in Supplementary Table S1.

**FIGURE 4:**
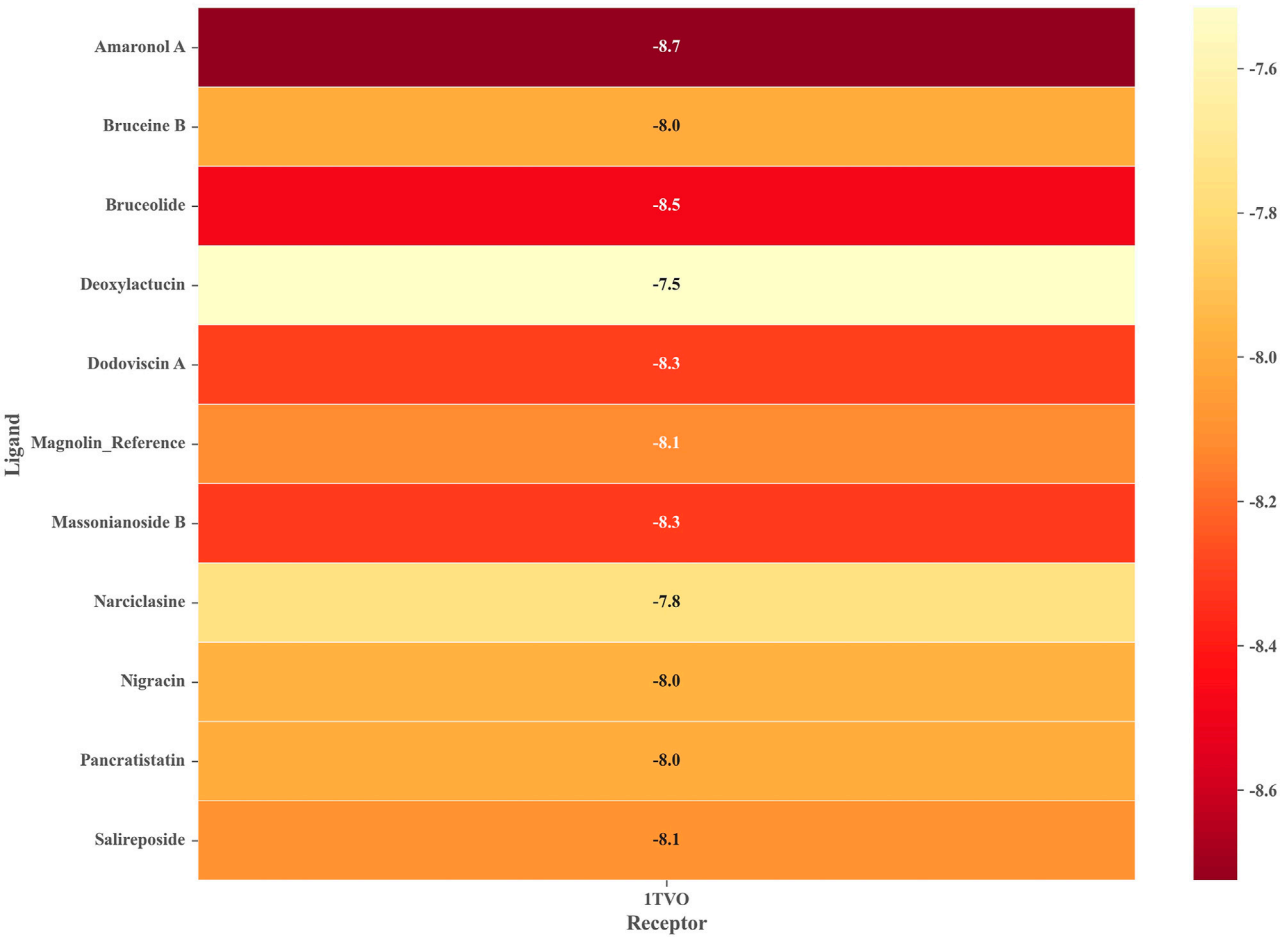
Binding free energy of 10 candidate compounds to ERK2 (PDB ID: 1TVO).

To ensure the reliability of the docking protocol, the co-crystallized ligand was re-docked to the active site of the target protein and the root mean square deviation (RMSD) between the docked conformation and the original conformation was calculated. The results showed a superimposed RMSD of 0.63 Å (less than 2 Å), which confirms the dependability of the docking process used in this study (Supplementary Figure S2). We next performed docking calculations using the same parameters for the 10 drug-like compounds mentioned above, whose binding free energies were shown in Figure 4. It should be noted that we chose magnolin, a natural product with ERK2 inhibitory activity, as an additional positive control molecule (Lee et al., 2015). We could find that all candidate compounds had binding free energies below −7.5 kcal/mol. Based on the principle that the lower the binding free energy, the stronger the ligand-receptor interaction, we screened four natural products with binding free energies below Magnolin (−8.125 kcal/mol) as potential ERK2 inhibitors. Specifically, Amaronol A exhibited the highest binding affinity (−8.725 kcal/mol), followed by Bruceolide (−8.488 kcal/mol) and finally Massonianoside B (−8.313 kcal/mol) and Dodoviscin A (−8.306 kcal/mol). The confidence levels of these four compounds in the machine learning models and the binding free energy in molecular docking were listed in Table 2. We could find that the confidence levels of compound Dodoviscin A were highest in both integrated models, while those of compound Bruceolide were relatively low.

**TABLE 2:** Details of 4 potential ERK2 inhibitors.

Compound name	Chemical structure	Binding free energy (kcal/mol)	Confidence level of voting model	Confidence level of stacking model
Amaronol A	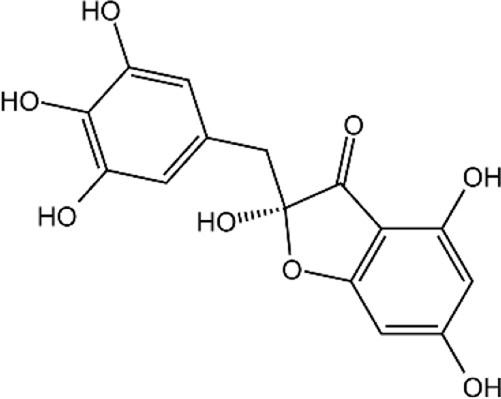	−8.725	0.786	0.831
Bruceolide	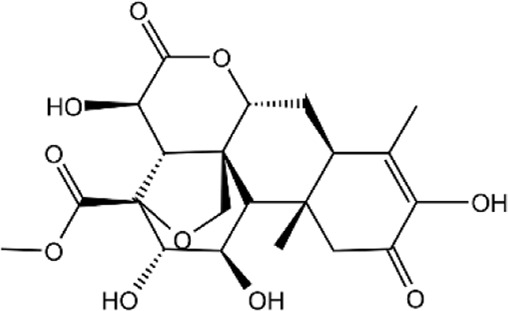	−8.488	0.781	0.772
Massonianoside B	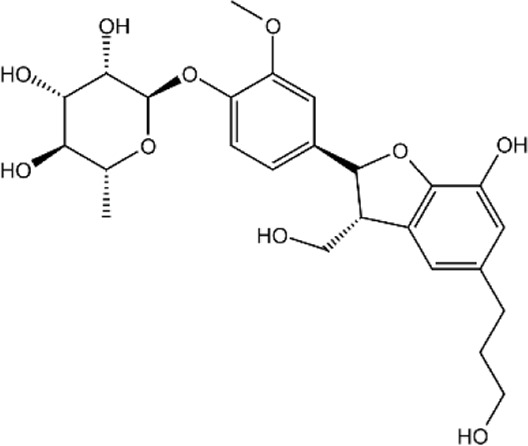	−8.313	0.794	0.843
Dodoviscin A	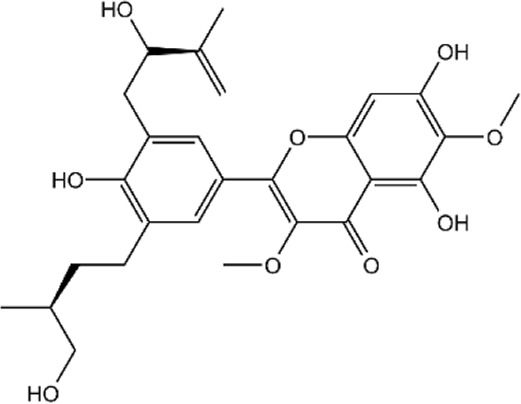	−8.306	0.807	0.845

### 3.4 Biological validation of the screened compounds

We retrieved the relevant reports of these four screened compounds in the PubMed database, of which there was little literature on Bruceolide and Amaronol A, and their *in vitro* biological activity was also unclear; while Dodoviscin A could inhibit melanogenesis and Massonianoside B had potential antioxidant activity (Chen et al., 2012; Yan et al., 2013). Therefore, Dodoviscin A and Massonianoside B were selected for the kinase assay given the application values. To determine the ERK2 inhibitory effect of those screened compounds, we performed the kinase activity assay based on the Lance^®^ Ultra method. The dose-response curves were shown in Supplementary Figure S3. At a concentration of 50 μm, Dodoviscin A exhibited 87.3% inhibition of ERK2 with an IC_50_ value of 10.79 μm. In contrast, Massonianoside B showed relatively weak inhibition of ERK2 with an IC_50_ value greater than 100 μm. These results confirmed the reliability of our virtual screening strategy.

### 3.5 Analysis of the binding pattern of screened compounds to target protein

**FIGURE 5:**
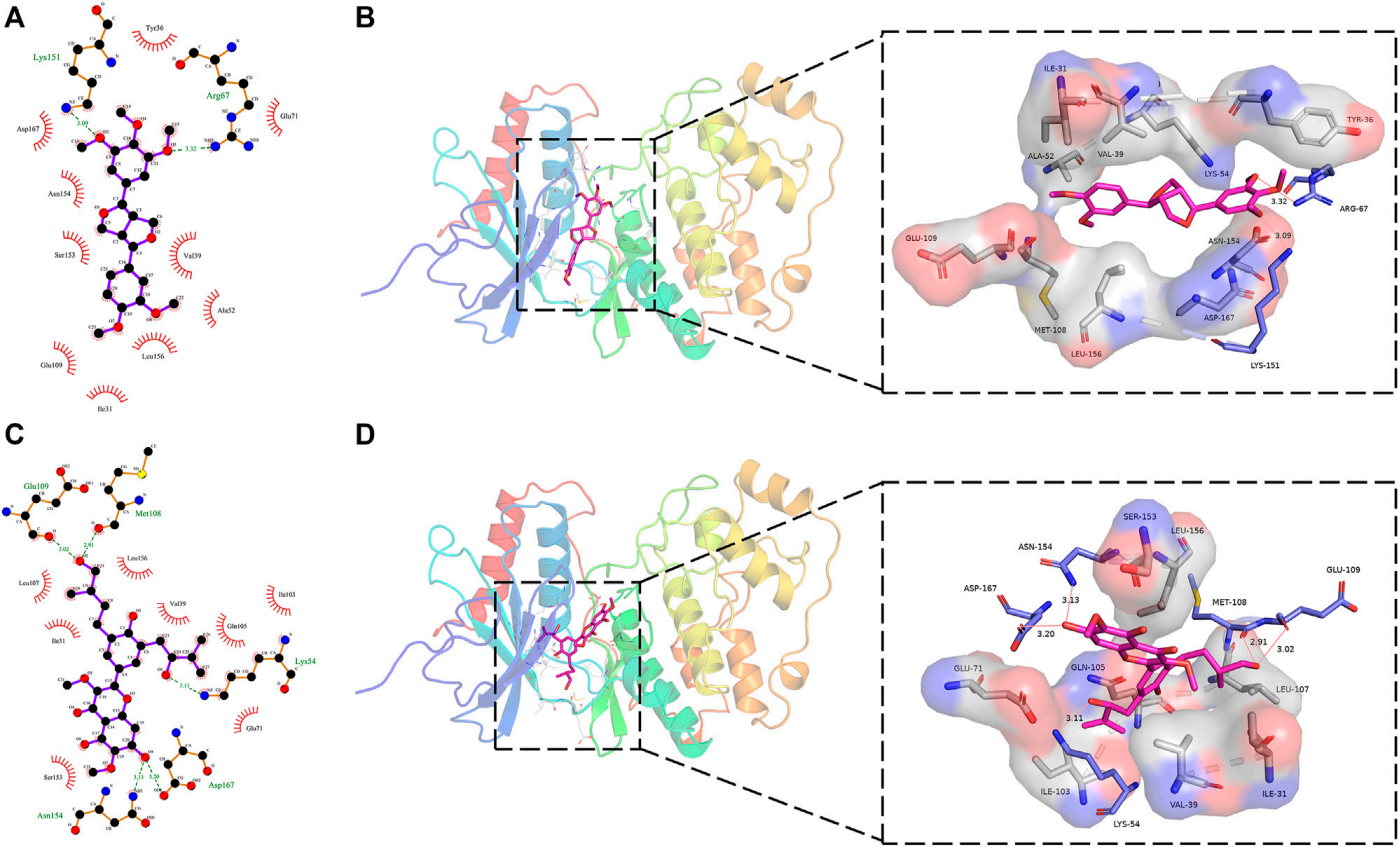
**(A,B)** 2D/3D binding interaction of Magnolin with ERK2 protein; **(C,D)** 2D/3D binding interaction of Dodoviscin A with ERK2 protein (pink structure represents small molecule ligand, blue structure represents hydrogen bond interaction residue, gray structure represents hydrophobic interaction residue).

Figure 5 showed the interaction of the target protein ERK2 with the positive control molecule Magnolin and the active candidate Dodoviscin A. We can see that both compounds docked to the hinge region of the kinase. Magnolin formed hydrogen bonds with amino acid residues LYS-151 and ARG-67 with bond lengths of 3.09 Å and 3.32 Å, respectively, while Dodoviscin A showed more hydrogen bonding interactions. Specifically, the key amino acid residues MET-108 and GLU-109 in the hinge region interacted with the hydroxymethyl group in the ligand structure *via* two hydrogen bonds of 2.51 Å and 3.02 Å, while three other amino acid residues, LYS-54, ASP-167, and ASN-154, were also observed to form hydrogen bonds with Dodoviscin A with bond lengths of 3.11 Å, 3.20 Å, and 3.13 Å, respectively. In addition, several amino acid residues including VAL-39, LEU-156, GLU-71, and SER-153 were involved in the formation of hydrophobic interactions. Previous studies have shown that hydrogen bonding interactions between type I inhibitors targeting ERK2 and MET-108 are critical for their occupancy of the ATP binding pocket (Roskoski, 2019). By comparing the binding patterns of these two compounds, we speculate that the formation of more hydrogen bonding interactions between Dodoviscin A and the target protein may account for its relatively high binding affinity.

### 3.6 Analysis of the binding stability of the screened compounds to the target protein

**FIGURE 6:**
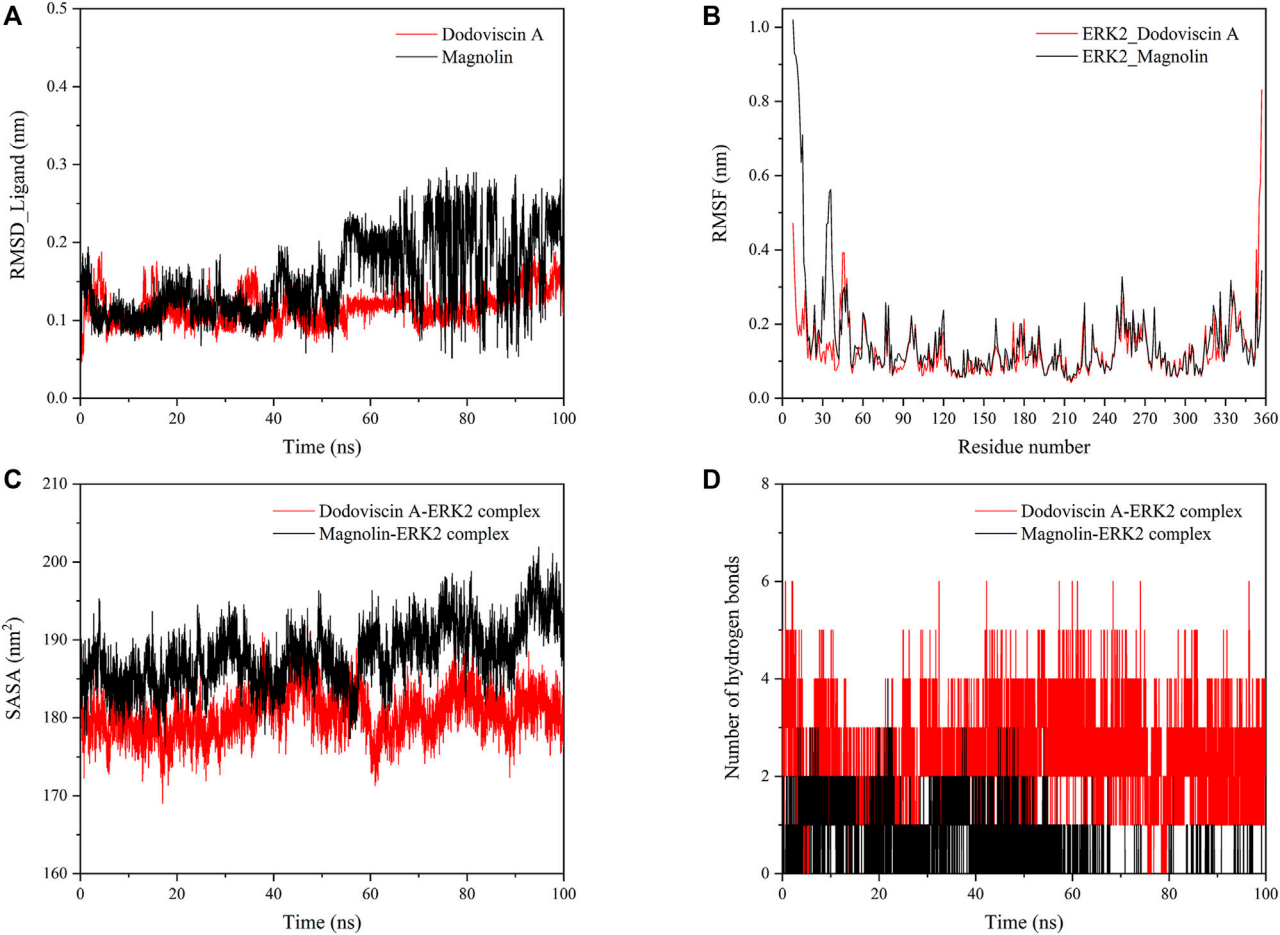
**(A)** Variation of RMSD values of small molecule ligands during molecular dynamics simulation; **(B)** Variation of RMSF values of protein amino acid residues during molecular dynamics simulation; **(C)** Variation of SASA values of ligand-protein complex during molecular dynamics simulation; **(D)** Variation of hydrogen bond number in ligand-protein complex during molecular dynamics simulation.

To further explore the dynamic binding process of the screened compound to the target protein, molecular dynamics simulations were performed. The RMSD values of the protein and ligand can be calculated to detect the change in the position of both during the simulation time, which is crucial for the stability of the complex in the dynamic system. In general, a low RMSD value indicates that there is little change in the conformation, while a less fluctuating RMSD value indicates that the whole system has reached stability (Raju et al., 2022). As shown in Figure 6A, the ligand compound Dodoviscin A bound to the receptor protein ERK2 reached equilibrium after initial fluctuations and remained there until the end of the simulation, with mean RMSD values of 0.116 ± 0.020 nm, while the mean RMSD value of Magnolin was 0.153 ± 0.050 nm. Notably, for the Dodoviscin A-ERK2 complex, although the mean RMSD values were not considerably different from those of the control Magnolin-ERK2 complex, the RMSD fluctuation trajectory was smoother and the binding stability was better.

Root Mean Square Fluctuations (RMSF) are commonly used to assess the flexibility state of the structural regions of proteins. As shown in Figure 6B, the receptor protein ERK2 bound to the ligand compounds Magnolin and Dodoviscin A had similar fluctuation trends, and most of the amino acid residues that were more flexible were those that formed interactions with the ligands.

**FIGURE 7:**
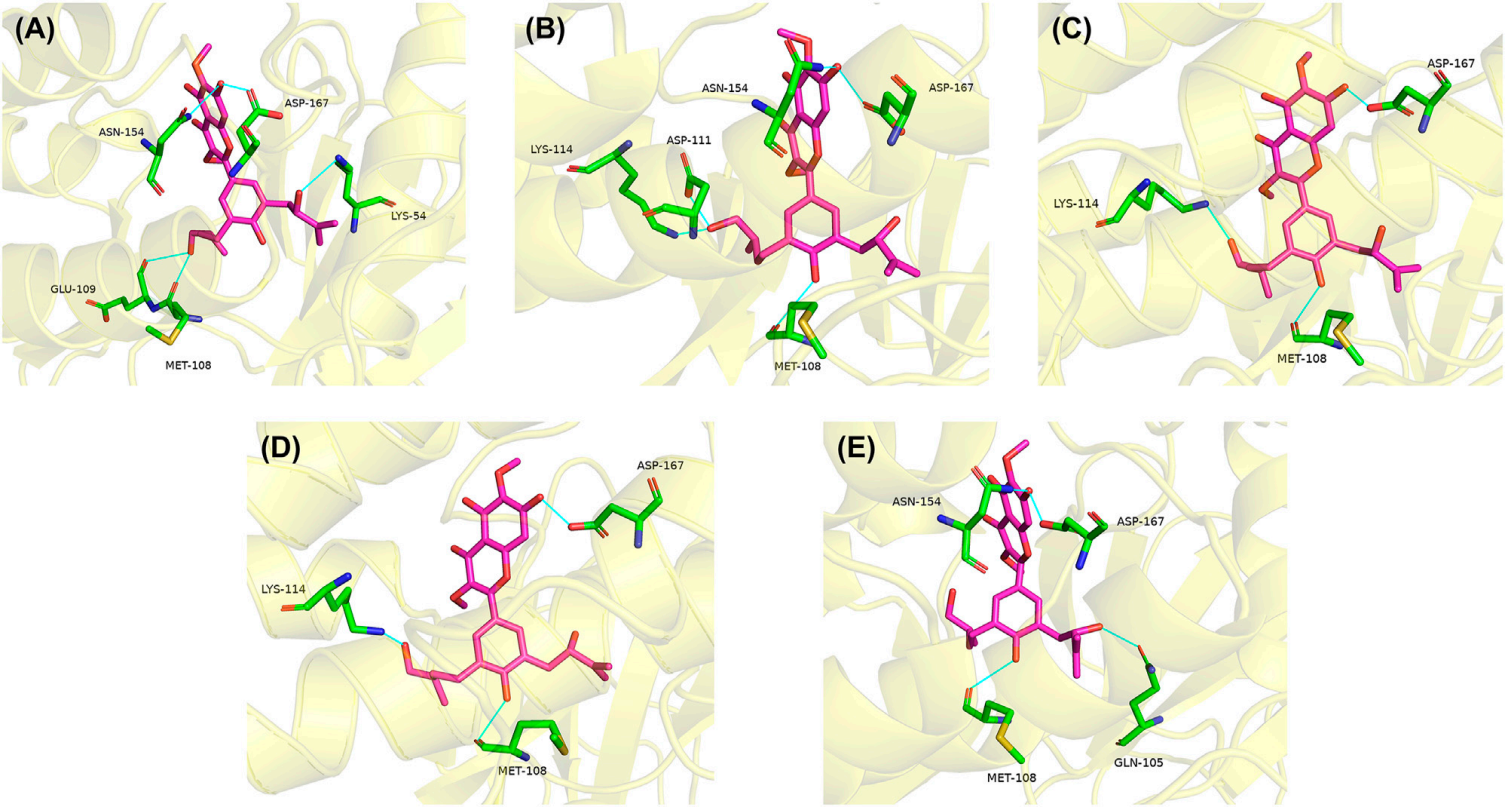
Analysis of hydrogen bonding interactions of Dodoviscin A-ERK2 complex at different time intervals during 100 ns simulation. **(A)** 10 ns; **(B)** 25 ns; **(C)** 50 ns; **(D)** 75 ns; **(E)** 100 ns.

The hydrophobicity of amino acid residues in proteins is closely related to the folding of proteins. Figure 6C showed the solvent accessible surface area (SASA) changes of both Dodoviscin A and Magnolin systems during the simulation, which is crucial for the stability of hydrophobic interactions. In general, the fluctuations of both systems were similar, with the SASA of the Dodoviscin A-ERK2 complex fluctuating between 170 and 190 nm^2^ with a mean value of 180.46 ± 2.81 nm^2^, while the overall SASA of the Magnolin-ERK2 complex was higher with a mean value of 188.01 ± 3.89 nm^2^. These results indicated that the amount of hydrophobic amino acids hidden inside the protein did not very much. Hydrogen bonding has a significant effect on the stable binding of ligand-protein complexes (Kuhn et al., 2010). Figure 6D displayed the variation in the number of hydrogen bonds during the simulation. We found that more hydrogen bonds were formed in the Dodoviscin A-ERK2 complex than in the control Magnolin-ERK2 complex, with up to six hydrogen bonding interactions observed, mainly between 2 and 4. To further observe the hydrogen bonding of the candidate compound Dodoviscin A to the target protein ERK2 during the 100 ns simulation, the ligand-protein interactions were visualized at different time intervals. The results were shown in Figure 7, compared to the initial conformation, the hydrogen bonds between Dodoviscin A and the two key amino acid residues MET-108 and ASP-167, which are more important for the binding of the inhibitor to ERK2, were still present despite the partial loss of hydrogen bonding interactions. The hydrogen bonding interactions of the Dodoviscin A-ERK2 complex at different time points were listed in Supplementary Table S2. These results also further illustrated that the candidate compound Dodoviscin A was able to bind steadily to the target protein ERK2.

## 4 Discussion

The human protein kinase family has become one of the hottest drug targets in the last few decades due to the critical role that mutations and dysregulation of protein kinases play in the pathogenesis of many human diseases (Cohen and Alessi, 2013). The Ras-Raf-MEK-ERK pathway is the most classical signaling pathway in the MAPK family, which transmits upstream signals to downstream responsive molecules through a continuous phosphorylation process. Studies have shown that inhibitors against the downstream target ERK have shown satisfactory results in terms of activity, selectivity and especially drug resistance compared to inhibitors against the upstream target (Miao and Tian, 2020). It should be noted that most of the ERK inhibitors identified so far are ATP-competitive inhibitors, and the development of more attractive covalent inhibitors still faces many challenges.

In recent years, the application of artificial intelligence in the pharmaceutical field has been increasing (Aguiar-Pulido et al., 2013). In this study, we first constructed multiple machine learning classification models for the kinase target ERK2 to discover potential natural product inhibitors. In contrast to previous work that considered only a single form of molecular characterization, we employed MACCS molecular fingerprints and RDKit molecular descriptors to provide a more comprehensive characterization of compounds in the dataset. Moreover, considering the variability between different algorithms, we have further improved the reliability of the predictions by integrating three single models through two model fusion strategies (Voting and Stacking). The results showed that our integrated models based on molecular descriptors performed better on the test set, with accuracy and AUC values ranging from 0.930 to 0.931 and 0.980–0.982, respectively. We then performed the first round of virtual screening of natural products in the ZINC database using these two integrated models, yielding a total of 403 candidate inhibitors.

The second round of virtual screening based on drug-likeness was carried out using the ADMETlab 2.0 online server, resulting in a total of 10 candidate inhibitors. Subsequently, we ranked the binding affinity of these compounds to ERK2 by Autodock Vina software (the third round of virtual screening) and identified the four most promising candidate inhibitors. Following *in vitro* biological evaluation, we found that the compound Dodoviscin A (a flavonoid isolated from Dodonaea viscosa) exhibited acceptable inhibitory activity on ERK2 (IC_50_ = 10.79 μm). In addition, a preliminary analysis of the interaction pattern and dynamic binding properties between the compound and the target protein was carried out. Overall, this work combines theoretical calculation and experimental validation in the search for natural product inhibitors of the kinase target ERK2, and the resulting candidate compound is expected to serve as a template molecule for the design of novel inhibitors.

The methods used in this study also have a number of limitations. Firstly, the data dependency and the lack of generalization may lead to a significant reduction in the performance of an excellent predictive model on a new dataset. Research has shown that an important issue to consider in building machine learning models is the quality of the dataset (Recanatini and Cabrelle, 2020). However, training data from various public databases is not always authentic and reliable, which will have a negative impact on the performance of machine learning models. Secondly, many machine learning algorithms are “black boxes” in terms of model interpretability. For example, we were unable to provide a detailed analysis of how the candidate compounds were screened. Thirdly, the number of compounds tested is relatively small and the screened compound Dodoviscin A still has considerable room for improvement in its inhibitory activity against ERK2. To address these issues, on the one hand, we need to clarify the potential of machine learning techniques to minimize prediction bias while understanding the nature of the problems (computational level), and on the other hand, we will work on structural modification of the screened compounds to improve their efficacy as ERK2 inhibitors (experimental level).

## Data Availability

The raw data supporting the conclusions of this article will be made available by the authors, without undue reservation.
