# Supplementary material for: Identification of potential extracellular signal-regulated protein kinase 2 inhibitors based on multiple virtual screening strategies
Source: Front Pharmacol. 2022 Nov 18;13:1077550. doi: 10.3389/fphar.2022.1077550 (PMC9715613; doi:10.3389/fphar.2022.1077550)
Supplement: Supplementary file 1 [file DataSheet1.docx]

Supplementary Material

# Supplementary Figures and Tables

## Supplementary Figures


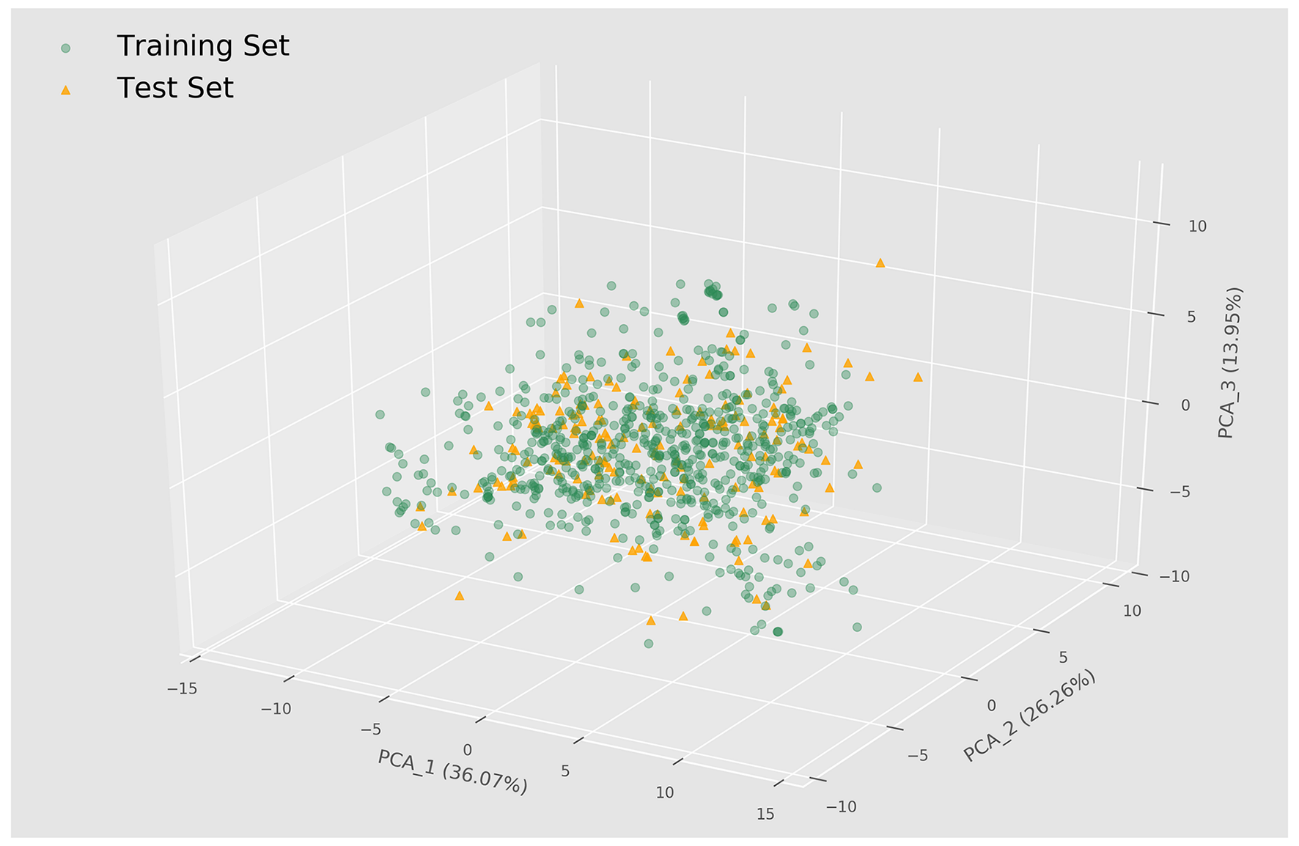


**Supplementary Figure 1.** Visualization of the three-dimensional distribution of training and test sets.


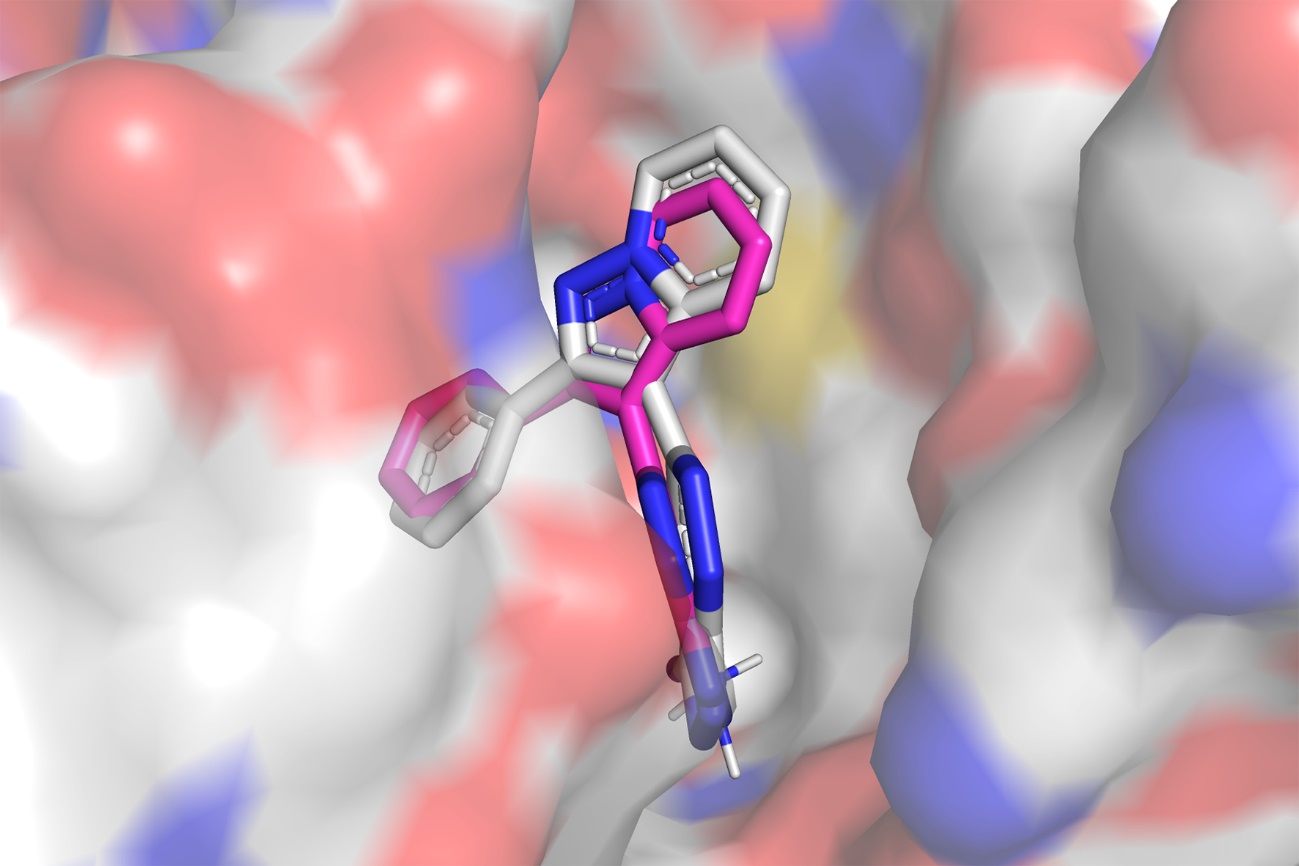


**Supplementary Figure 2.** The superimposition of the docked co-crystallized ligand (pink stick) with its X-ray crystal structure (gray stick).


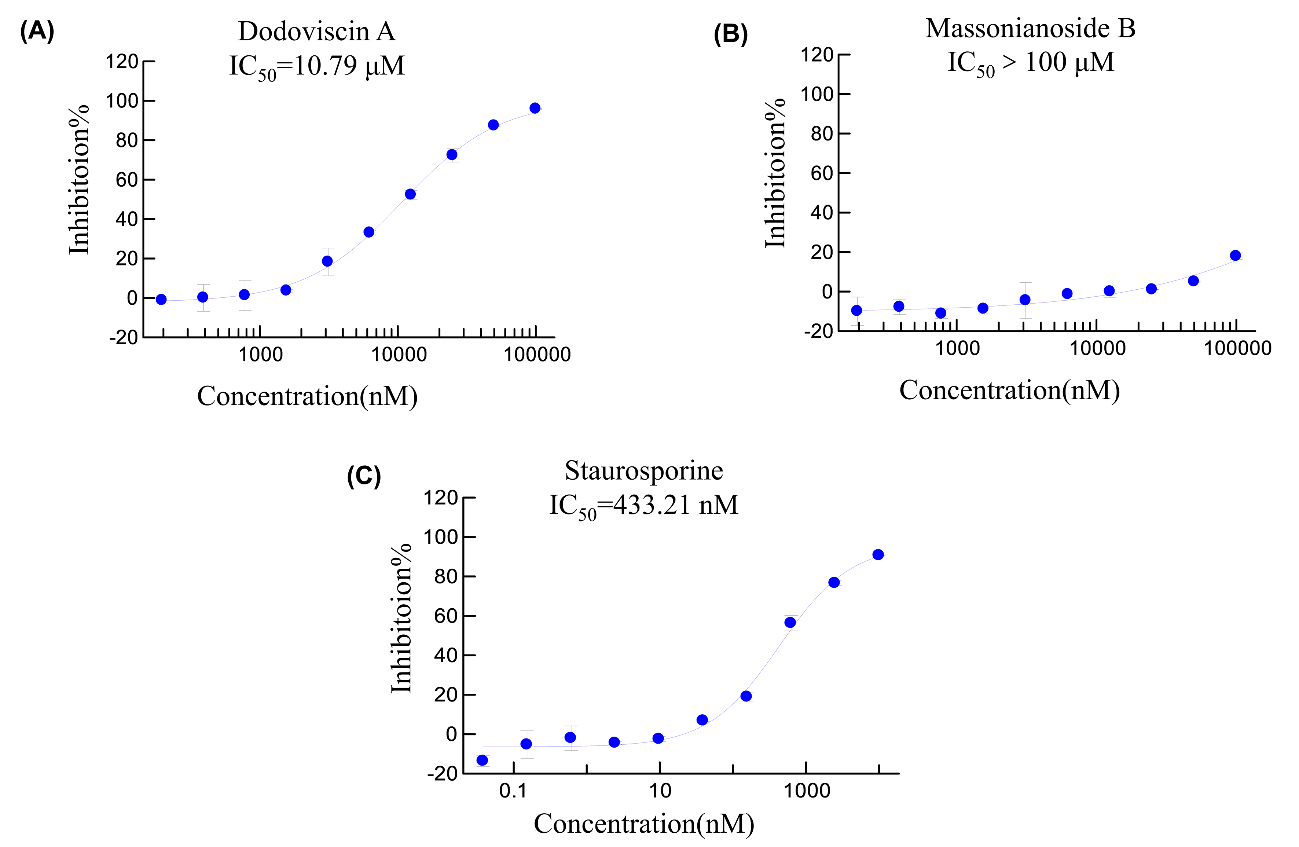


**Supplementary Figure 3.** (A) The dose-dependent inhibition curve of Dodoviscin A against ERK2; (B) The dose-dependent inhibition curve of Massonianoside B against ERK2. (C) The dose-dependent inhibition curve of the positive control against ERK2.

## Supplementary Tables

**Supplementary Table 1.** Partial physicochemical properties of the 10 candidate compounds obtained from the second round of screening

| Compound Name | Molecular Weight | LogP | LogS | Number of Hydrogen Bond Acceptors | Number of Hydrogen Bond Donors | Number of Hydrogen Bond Acceptors |
| --- | --- | --- | --- | --- | --- | --- |
| Dodoviscin A | 500.20 | 3.77 | -3.96 | 9 | 5 | 10 |
| Bruceine B | 480.16 | 0.39 | -3.92 | 11 | 2 | 4 |
| Massonianoside B | 492.20 | 1.13 | -3.90 | 10 | 6 | 8 |
| Narciclasine | 307.07 | -0.70 | -2.73 | 8 | 4 | 0 |
| Bruceolide | 438.15 | -0.04 | -3.28 | 10 | 3 | 2 |
| Pancratistatin | 325.08 | -0.66 | -0.83 | 9 | 6 | 0 |
| Salireposide | 406.13 | 1.05 | -2.34 | 9 | 5 | 7 |
| Deoxylactucin | 260.10 | 1.01 | -2.74 | 4 | 0 | 1 |
| Nigracin | 406.13 | 1.13 | -2.62 | 9 | 5 | 7 |
| Amaronol A | 320.05 | 1.46 | -2.34 | 8 | 6 | 2 |

**Supplementary Table 2.** Detailed information on the hydrogen bonding interactions of the Dodoviscin A-ERK2 complex at different time points during the simulation

| Time point | Amino acid residue | Distance | |
| --- | --- | --- | --- |
| 0 ns  25 ns  50 ns  75 ns  100 ns | LYS-54  MET-108  GLU-109  ASN-154  ASP-167  MET-108  ASP-111  LYS-114  ASN-154  ASP-167  MET-108  LYS-114  ASP-167  MET-108  LYS-114  ASP-167  GLN-105  MET-108  ASN-154  ASP-167 | 3.11 Å  2.91 Å  3.02 Å  3.13 Å  3.20 Å  3.12 Å  2.60 Å  2.82 Å  3.00 Å  2.62 Å  2.72 Å  2.90 Å  2.72 Å  2.95 Å  2.80 Å  2.59 Å  2.85 Å  3.16 Å  3.08 Å  2.56 Å |  |
